# Identification of culling reasons, intervals, and risk factors by postpartum period classification in dairy farms

**DOI:** 10.5713/ab.25.0343

**Published:** 2025-10-22

**Authors:** Hyun-Gu Kang, Jae-Kwan Jeong, Ill-Hwa Kim

**Affiliations:** 1 College of Veterinary Medicine, Chungbuk National University, Cheongju, Korea; 2 Warm Vet Clinic, Cheongju, Korea

**Keywords:** Body Condition, Culling Reason, Dairy Cow, Disorder, Period, Risk

## Abstract

**Objective:**

This study aimed to identify the reasons, intervals, and risk factors in Korean dairy farms where cows produce large volumes of milk under challenging conditions.

**Methods:**

A total of 11,361 calving datasets were analyzed to determine culling reasons and intervals, and the factors affecting culling risk. The postpartum period was classified into three categories: within 60 days (Period 1), between 61 and 210 days (Period 2), and beyond 210 days (Period 3) postpartum.

**Results:**

The culling rates were 7.4% in Period 1, 8.5% in Period 2, and 11.3% in Period 3, totaling 27.2%. The main reasons for culling were infertility, mastitis, voluntary causes, peripartum disorders, and other health problems. Culling due to infertility (333 days), mastitis (142 days), or other health problems (129 days) was associated with longer median calving-to-culling intervals, whereas culling due to peripartum disorders (19 days) had shorter intervals compared to voluntary culling (101 days, p<0.0001). Cows with a higher body condition score (BCS) (≥3.75) at dry-off were more likely to be culled in Period 1 (odds ratio [OR]: 1.83, p<0.0001) than those with a BCS of 3.5. In period 1, cows with a BCS increase between dry-off and calving had a lower likelihood of culling (OR: 0.65) than cows with no BCS change, while those with a BCS decrease had a higher likelihood of culling (OR: 1.29) in Period 2 (p<0.05). Cows with peripartum disorders were more likely to be culled throughout all periods (p<0.01) than cows without disorders. The probability of culling increased (p<0.0001) with higher parity across all periods.

**Conclusion:**

Preventing high BCS at dry-off and BCS reduction during the dry period, minimizing the incidence of peripartum disorders and other health problems, and improving reproductive efficiency can help reduce involuntary culling.

## INTRODUCTION

Culling and replacement must be well-coordinated to maintain optimal dairy herd turnover. Culling has been traditionally classified as involuntary (e.g., due to infertility, disorders, or death) and voluntary (e.g., low milk yield, surplus cows, or high parity) [1]. A higher rate of involuntary culling results in severe economic losses due to increased replacement heifer costs and reduced milk production from first-lactation cows [2,3]. Conversely, reducing the proportion of involuntary culling allows for greater flexibility in voluntary culling decisions, which can maximize herd productivity and contribute to the sustainability of dairy herds [4].

Numerous studies have investigated the incidence and reasons for culling in dairy herds worldwide [5–7]. This extensive research has shown that culling decisions are influenced by various factors, including cow fertility, health, productivity, climate, production and management systems, and owners’ policies [7–9]. As a result, culling rates vary between 21% and 36% across studies [10–12]. Although the specific reasons for culling differ slightly between studies, infertility and mastitis are consistently reported as the primary causes of involuntary culling, while low milk production and old age are common reasons for voluntary culling [5,13]. Moreover, the proportion of involuntary culling among total culling ranges from 68% to 92% [9,14,15]. This substantial variation likely contributes to significant differences in profitability across studies. In practice, the reasons for culling and the associated risk factors can vary depending on the region and country, as dairy management, production systems, and environmental conditions differ. In regions or countries with high milk production achieved through intensive genetic selection and production systems, the risk of involuntary culling increases due to higher incidences of health problems and reduced fertility [4,16].

It is of the utmost importance to identify the causes of culling and implement preventive measures. Additionally, understanding the risk factors associated with culling is essential for reducing involuntary culling [4,9,14]. In this study, we hypothesized that classifying the postpartum period according to the distinct physiological and management phases that dairy cows experience after calving, and identifying the associated risk factors for culling, along with analyzing actual culling causes based on culling intervals, would enhance preparedness for involuntary culling. Therefore, this study was conducted to identify the reasons and timing of culling, as well as the factors influencing culling risk over time after calving in dairy farms in midwestern South Korea.

## MATERIALS AND METHODS

### Participating farms and animals

This study involved 27 dairy farms in midwestern South Korea from 2010 to 2023. Each farm had between 25 and 260 lactating dairy cows, with an average of 61 cows. The cows were maintained in loose housing systems, fed total mixed rations, and milked twice a day. Milk yield and somatic cell counts were measured and recorded monthly by the Korean Animal Improvement Association. Mean annual milk yields ranged from 9,523 to 11,728 kg, and the mean somatic cell counts ranged from 121,400 to 451,200 cells/mL across the farms.

### Health and reproductive management

All cows on the participating farms underwent herd health and reproductive checks by the authors every 2 to 4 weeks. These checks included monitoring for health issues and examining the reproductive organs (ovaries and uterus) via ultrasonography with a 6.5 MHz array transducer (SR-1C-Wireless Rectal Scanner; SongKang GLC). Each follicle ≥8 mm in diameter was measured, and the presence or absence of a corpus luteum was also recorded. Simultaneously, the body condition scores (BCS) were evaluated using a five-point scale with quarter-point divisions [17]. Health problems defined in this study were based on those reported previously [18–23]. Among these, peripartum disorders, included as a variable in the analysis of culling risk, are described below, and other health disorders are listed in Table 1. Dystocia was defined as difficult calving requiring assistance. Retained placenta was defined as retention of the fetal membranes for longer than 24 h. Septicemic metritis was described as having a fever and watery, fetid, red-brown uterine discharge. Ketosis was diagnosed as having anorexia and depression, and acetone-like odor on the breath. Milk fever was diagnosed as having nervousness, weakness, and inability to rise following calving. Abomasal displacement was diagnosed by abdominal auscultation with a characteristic ping sound. Clinical endometritis was diagnosed based on the occurrence of a mucopurulent uterine discharge and ultrasonography.

For the reproductive management in participating farms, the voluntary waiting period from calving to the first artificial insemination (AI) ranged from 40 to 60 days, depending on the individual farm. In addition to estrus detection, various herd reproductive programs were implemented, including Ovsynch, Presynch-Ovsynch, Double-Ovsynch, and modified Presynch-Ovsynch protocols [24–26]. Pregnancy diagnosis was performed via transrectal ultrasonography at 30 and 60 days after AI.

### Data collection and study design

A total of 11,361 calving datasets were collected from 27 dairy farms in the midwestern region of South Korea between 2010 and 2023. All data were obtained from the geographic location around 36.7821° N latitude and 126.4522° E longitude. The datasets included information on parity, dates of reproductive events (calving, AI, and pregnancy diagnosis), BCS at dry-off and calving, peripartum disorders, other health problems, and the dates and reasons for culling (Table 2). By analyzing the accumulated datasets from herd health and reproductive checks at each farm, this study identified: 1) the culling reasons and intervals and 2) the factors affecting culling risk by classifying the postpartum period as follows: within 60 days (the voluntary waiting period, Period 1), between 61 and 210 days (the intensive breeding period, Period 2), and beyond 210 days (the late lactation/dry period, Period 3) postpartum.

### Statistical analysis

Results are expressed as means±standard error of the mean, with some exceptions in descriptive statistics (means±standard deviation). For statistical analysis, cows were categorized by parity (1, 2, 3, 4, 5, and ≥6); by BCS at dry-off (≤3, 3.25, 3.5, and ≥3.75); by BCS change between dry-off and calving (no change, increase, or decrease); and by the occurrence of peripartum disorders (no or yes). Calving season was categorized as spring (March to May), summer (June to August), autumn (September to November), or winter (December to February). All analyses were performed using SAS statistical software (ver. 9.4, SAS Institute).

A Cox proportional hazards model using the PHREG procedure was used to compare the likelihood of culling within 400 days postpartum based on the main reasons for culling (infertility, mastitis, voluntary causes, peripartum disorders, and other health problems). This analysis included 2,790 calving datasets from cows culled for these reasons and yielded an estimate of the likelihood of a cow being culled at a given time. The time used in this model was the number of days between calving and culling. The model included parity (1 to ≥6), calving season, the main reasons for culling, and interactions between these variables. Cow, year, and farm were included as random effects in the model. The proportional hazards were determined by analyzing interactions between explanatory variables and time and evaluating Kaplan-Meier curves. The median and mean number of days to culling were determined by survival analysis in the Kaplan-Meier model using the LIFETEST procedure within the SAS software. A survival plot was generated using the Survival option within MedCalc software (ver. 11.1; MedCalc Software).

Risk factors for culling were analyzed using logistic regression with the GLIMMIX procedure and the odds ratio (OR) option, classifying outcomes by postpartum period (Periods 1, 2, and 3). Of the 11,361 calving datasets, 1,511 (13.3%) were excluded because they did not meet all the required variables for analyzing the risk of culling. Thus, 9,850 datasets that fully met the necessary variables were included in the analyses. The logistic regression model included BCS at dry-off (≤3, 3.25, 3.5, and ≥3.75), BCS change during the dry period (no change, increase, or decrease), history of peripartum disorders (no or yes), parity (1, 2, 3, 4, 5, and ≥6), and calving season. Cow, year, and farm were included as random effects in the model. ORs and 95% confidence intervals were calculated. A p-value<0.05 was considered significant, and 0.05 ≤ p<0.1 was considered to indicate a tendency toward a significant difference.

## RESULTS

### Descriptive statistics on the reasons for culling

Among the 11,361 cows that calved, 3,091 (27.2%) were culled, with culling rates from 17.9% to 36.5% across the 27 farms. The culling rates were 7.4% in Period 1, 8.5% in Period 2, and 11.3% in Period 3. Table 2 presents descriptive statistics for the reasons for culling. Of the 3,091 culled cows, 86.6% were removed from their farms, while 13.4% died. Additionally, 75.5% were culled for involuntary reasons and 18.5% for voluntary reasons.

The reasons for culling were infertility (27.8%), mastitis (20.5%), voluntary causes (18.5%), peripartum disorders (14.1%), other health problems (9.3%), digestive disorders (1.9%), locomotor problems (1.0%), infectious diseases (0.9%), and unknown causes (6.0%). The overall mean parity of the culled cows was 3.0±1.7.

### Likelihood of culling within 400 days postpartum and culling intervals by main reasons for culling

Table 3 presents the factors affecting the likelihood of culling within 400 days postpartum, as determined using Cox’s proportional hazard model. The hazard of culling within 400 days postpartum decreased in cows culled due to infertility (hazard ratio [HR]: 0.28, p<0.0001), mastitis (HR: 0.85, p<0.01), or other health problems (HR: 0.86, p<0.05) compared to those culled due to voluntary causes. Conversely, the hazard increased in cows culled due to peripartum disorders (HR: 2.25, p<0.0001) than in those culled due to voluntary causes.

**Figure 1:**
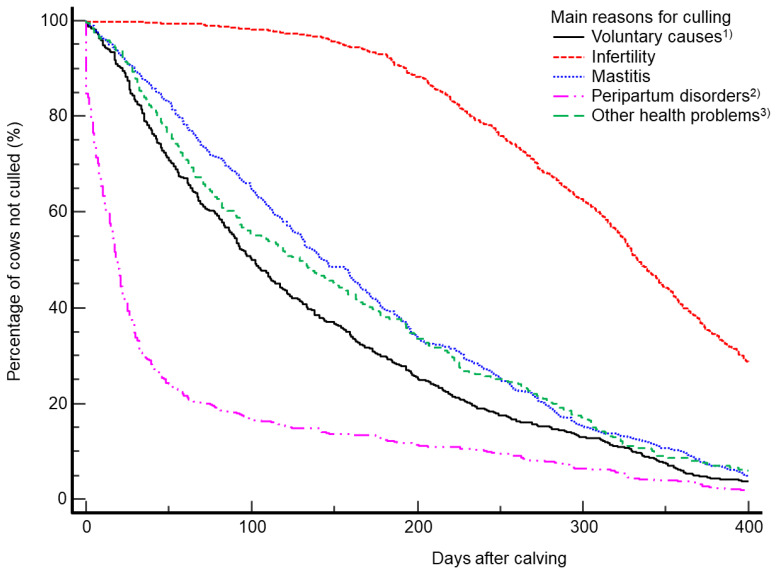
Survival curves generated using MedCalc software for the interval between calving and culling in dairy cows culled due to voluntary causes (n = 573), infertility (n = 860), mastitis (n = 635), peripartum disorders (n = 435), or other health problems (n = 287). The median and mean days between calving and culling were longer (p<0.0001) in cows culled due to infertility (333 and 315.2±2.9), mastitis (142 and 166.9±4.6), or other health problems (129 and 157.5±7.1), but shorter in cows culled due to peripartum disorders (19 and 59.9±4.8) compared to cows culled due to voluntary causes (101 and 136.3±4.7). ^1)^ Voluntary causes indicate high parity, low milk yield, bad temperament, high somatic cells, udder problems, and overstock. ^2)^ Peripartum disorders indicate dystocia, retained placenta, uterine rupture, uterine or vaginal prolapse, septicemic metritis, uterine adhesion, cervicitis, pyometra, ketosis, milk fever, abomasal displacement, and clinical endometritis. ^3)^ Other health problems indicate anemia, fracture, peritonitis, sinusitis, traumatic reticuloperitonitis, pneumonia, cardiovascular diseases, heat stress, shock, and tumors.

The median and mean days between calving and culling were longer (p<0.0001) in cows culled due to infertility (333 and 315.2±2.9), mastitis (142 and 166.9±4.6), or other health problems (129 and 157.5±7.1), but shorter culled due to peripartum disorders (19 and 59.9±4.8) compared to those culled due to voluntary reasons (101 and 136.3±4.7), as shown by the survival curves (Figure 1).

In addition, the hazard of culling within 400 days postpartum tended to increase in cows with parity 2 (HR: 1.13, p<0.1) and increased linearly in cows with a parity of 3 to ≥6 (HR: 1.16 to 1.73, p<0.05 to 0.0001), compared to primiparous cows. Cows that calved during the summer (HR: 1.12, p<0.1) were more likely to be culled within 400 days postpartum than those that calved during the spring.

### Risk factors for culling by time period after calving

Table 4 shows the factors affecting the risk of culling in Periods 1, 2, and 3, using logistic regression in the GLIMMIX procedure. Cows with a higher BCS (≥3.75) at dry-off were more likely to be culled (OR: 1.83, p<0.0001) in Period 1 than those with a BCS of 3.5. Cows with a BCS increase between dry-off and calving had a lower likelihood of being culled (OR: 0.65, p<0.05) in Period 1 than those without BCS change. Conversely, cows with a BCS decrease during the dry period had a higher likelihood of being culled (OR: 1.29, p<0.05) in Period 2. Cows with peripartum disorders were more likely to be culled throughout all periods (ORs: 1.26 to 1.65, p<0.01 to<0.0001) compared to cows without these disorders. The probability of culling increased (p<0.0001) with higher parity across all periods. In period 1, cows with parity of 3 to ≥6 showed higher culling likelihood (ORs: 1.94 to 5.69) compared to primiparous cows. Similarly, in Periods 2 and 3, the culling likelihood increased in cows with parity 2 to ≥6 (Period 2 ORs: 2.08 to 5.86; Period 3 ORs: 1.63 to 2.87) compared to primiparous cows. In contrast, the calving season did not significantly affect the probability of culling in any of the three periods (p>0.05).

## DISCUSSION

This study showed that the most prevalent reasons for culling were infertility and mastitis, followed by voluntary causes and peripartum disorders. Culling due to infertility, mastitis, or other health problems was associated with longer median calving-to-culling intervals, whereas culling due to peripartum disorders had shorter intervals compared to voluntary culling. The history of peripartum disorders and increasing parity were risk factors for culling throughout all periods (Periods 1, 2, and 3), while a high BCS (≥3.75) at dry-off was a risk factor for culling in Period 1. In addition, a BCS increase during the dry period reduced the likelihood of culling in Period 1, while a BCS decrease increased the likelihood of culling in Period 2, indicating that the effect of BCS on culling varies over time postpartum.

The overall culling rate in the present study was 27.2%, although there was a big difference between farms, ranging from 17.9% to 36.5%. The overall culling rate observed in this study falls within the range reported in previous studies (21.3% to 36.3%; [10–12]). Differences in culling rates among studies may result from the complexity of factors influencing culling decisions, including milk production levels, variations in production and management systems, climate, and owners’ culling policies [3,7,27]. Nevertheless, the optimal culling rate (or herd turnover rate) has been suggested to be below 30% [1]. The proportion of involuntary culling (75.5%) in this study was similar to that reported in a previous study (73%; [28]), although the range varies widely (68% to 92%; [9,14,15]). Reducing involuntary culling and increasing voluntary culling are critical for maintaining an optimal herd turnover. Therefore, the lower proportion of voluntary culling (18.5%) observed in this study compared to the 23.4% reported in another study [29] has significant implications, as it may have negatively affected herd profitability.

In the present study, infertility was the most prevalent reason for culling, followed by mastitis. However, other studies have reported that while infertility was the common reason for culling, as in our study, low milk production was the second most common [2,29], partially differing from our findings. Additionally, a Danish study reported that milk production issues and reproduction problems were the most frequent reasons for culling [30]. This discrepancy may reflect a different culling management strategy in Korea, where voluntary culling, such as reduced milk production, tends to be delayed as much as possible. Additionally, peripartum disorders and other health problems were important culling reasons in this study, as seen in other reports [2,15,31]. These findings highlight the importance of implementing preventive measures against mastitis and peripartum disorders during the transition period and of minimizing the delay between calving and conception through active reproductive programs.

Our study showed that culling due to infertility, mastitis, or other health problems extended the culling interval after calving. Conversely, culling due to peripartum disorders shortened the culling interval after calving compared to voluntary causes. These observations are reasonable because many peripartum disorders occur during early lactation, as reported in previous studies [32,33]. Furthermore, these disorders are often more life-threatening than infertility, mastitis, or other health problems. Infertility resulted in the longest culling interval after calving among the culling reasons in the present study, consistent with findings from several studies [31,32,34].

This study examined risk factors for culling by classifying them over time after calving. Our finding that a higher BCS (≥3.75) at dry-off was a significant risk factor for culling in Period 1 is likely due to the increased incidence of the peripartum disorders among cows with high BCS (69.7% [≥3.75] vs. 22.3% [≤3.5] in this study), likely resulting from more severe BCS loss during the dry period (0.6±0.02 [≥3.75] vs. 0.20±0.01 [≤3.5]), as previously suggested [35]. The lack of an effect of high BCS at dry-off on culling in Periods 2 or 3 may be because its impact is limited to early lactation. Moreover, our finding that a BCS increase during the dry period reduced the likelihood of culling in Period 1, while a BCS decrease increased it in Period 2, highlights the importance of nutritional management during the dry period in predicting subsequent culling risk. We found that BCS loss during the dry period did not influence culling in later lactation, similar to the pattern observed with high BCS at dry-off. Studies investigating BCS changes during the dry period as a culling risk factor are limited. One study reported that among cows with a normal dry period, those with excessive BCS loss were less likely to be culled within 60 days postpartum compared to cows with moderate BCS loss, no change, or gain [18]. This study also found that cows with excessive BCS loss had a reduced hazard of being removed from the herd by 305 days postpartum compared to cows that gained BCS. In contrast, cows with no BCS change had a higher risk of removal than those that gained BCS [18]. These findings differ from ours. Another study reported that cows with a low BCS (<3.0) during the first month after calving were at a greater risk of culling than those with a better BCS (≥3.0) [36]. The effects of BCS changes during the dry period on culling in Periods 1 and 2 in this study are likely related to energy balance status during the transition period. Severe BCS loss may lead to negative energy balance, predisposing cows to metabolic disorders and health issues due to decreased immunity [37]. Conversely, an increase in BCS during the dry period may be beneficial, possibly preventing peripartum disorders by maintaining positive energy balance and good health. Therefore, based on our findings, preventing high BCS at dry-off and minimizing BCS reduction during the dry period, regardless of the initial BCS, should be emphasized in herd management.

The history of peripartum disorders and increasing parity were identified as risk factors for culling in Periods 1, 2, and 3. Our finding that peripartum disorders are a risk factor for culling throughout the postpartum period suggests that these conditions may directly increase the likelihood of culling. This is likely because such disorders negatively impact cow welfare, cause pain, and can become more severe, potentially leading to death. Alternatively, peripartum disorders may indirectly contribute to culling by impairing subsequent reproductive performance through carry-over effects. Similarly, several studies have shown that peripartum disorders, including dystocia, retained placenta, metabolic disorders, and other disorders, negatively affect reproductive performance in dairy cows [31,38]. Consistent with our results, previous studies have also reported that peripartum disorders were significant risk factors for culling in dairy herds [8]. These findings indicate that disorders occurring within the first month postpartum have consistently been associated with culling risk. Therefore, the prevention of peripartum disorders and the prompt, proper treatment of affected cows are crucial for herd health and productivity.

Our finding that increasing parity is a risk factor for culling across all periods also aligns with previous studies [6,30]. Several studies have reported that aging increases the risk of culling, likely due to high milk yield, severe BCS loss during early lactation, weakened immune defenses, a higher risk of peripartum disorders, and reduced reproductive efficiency [39,40]. In the present study, the calving season did not significantly affect the probability of culling in any period. However, unlike our findings, studies conducted in pasture-based systems have shown that many infertile cows are culled during the autumn grazing season to reduce feeding costs before the housing period [10]. Another study reported that the risk of culling due to death was higher in cows that calved during the spring and summer [33].

Based on the findings in this study, farm management strategies to reduce involuntary culling can be summarized as follows. First, it is essential to minimize the occurrence of infertility and mastitis, which were identified as direct causes of involuntary culling. At the same time, preventing excessive BCS at dry-off and minimizing BCS loss during the dry period are crucial to reduce the risk of peripartum disorders, which were found to be important risk factors for culling. Furthermore, improving reproduction efficiency through proactive reproductive management, including the use of synchronization protocols, may contribute to lowering involuntary culling rates.

## CONCLUSION

Summarizing the risk factors identified for culling over time after calving, along with the actual causes of culling based on culling intervals in the present study, it is evident that prepartum BCS status and changes, peripartum disorders, and reduced reproductive efficiency after calving are interrelated and collectively associated with an increased risk of culling. Therefore, preventing high BCS at dry-off, minimizing BCS loss during the dry period, reducing the incidence of peripartum disorders and other health problems, and enhancing reproductive efficiency through active reproductive programs may help reduce involuntary culling in dairy farms.

## Figures and Tables

**Table 1:** Classification of culling reasons of 3,091 dairy cows from 27 farms in midwestern South Korea

Category	Definition/detailed items
High parity	A parity of 4 or ≥5, possibly associated with secondary problems in some cases
Low milk yield	Insufficient milk production as determined by the owner’s judgment
High somatic cells	Long-lasting high somatic cell count, as judged by the owner
Udder problems	Morphological defects in the udder
Overstock	Excessive number of cows beyond farm capacity
Infertility	Failure to conceive, with some cases involving abortion, maceration, or mummification of fetuses
Mastitis	Severe clinical mastitis not responding to treatment
Digestive disorders	Melena, HBS, enteritis, abomasal ulcer, bloat, and intestinal torsion
Peripartum disorders	Dystocia, retained placenta, uterine rupture, uterine or vaginal prolapse, septicemic metritis, uterine adhesion, cervicitis, pyometra, ketosis, milk fever, abomasal displacement, and clinical endometritis
Locomotor problems	Lameness, arthritis, foot rot, and hip dislocation
Infectious diseases	Bovine leukosis and tuberculosis
Other health problems	Anemia, fracture, peritonitis, sinusitis, TRP, pneumonia, cardiovascular diseases, heat stress, shock, and tumors
Unknown reasons	Not recorded

HBS, hemorrhagic bowel syndrome; TRP, traumatic reticuloperitonitis.

**Table 2:** Descriptive statistics on the reasons for culling 3,091 dairy cows from 27 farms in midwestern South Korea

Reasons for culling	Removal		Death		Combined removal and death		
		
	No. cows	Percentage of cows (%)	No. cows	Percentage of cows	No. cows	Percentage of cows (%)	Mean parity (mean±SD)
Voluntary causes							
High parity	242	7.8	-	-	242	7.8	5.5±1.4
Low milk yield	129	4.2	-	-	129	4.2	2.2±1.2
Bad temperament	57	1.8	-	-	57	1.8	2.7±1.2
High somatic cells	58	1.9	-	-	58	1.9	3.5±1.7
Udder problems	42	1.3			42	1.3	3.2±1.5
Overstock	45	1.5	-	-	45	1.5	2.1±1.0
Subtotal voluntary causes	573	18.5	-	-	573	18.5	3.8±2.0
Involuntary causes							
Infertility	860	27.8	-	-	860	27.8	2.7±1.6
Mastitis	611	19.8	24	0.7%	635	20.5	3.0±1.5
Peripartum disorders	268	8.7	167	5.4%	435	14.1	3.2±1.6
Digestive disorders	27	0.8	33	1.1%	60	1.9	2.7±1.4
Locomotor problems	31	1.0	-	-	31	1.0	3.3±1.7
Infectious diseases	27	0.9	-	-	27	0.9	3.1±1.7
Other health problems	103	3.3	184	6.0%	287	9.3	2.7±1.5
Subtotal involuntary causes	1,927	62.3	408	13.2%	2,335	75.5	2.9±1.6
Unknown causes	178	5.8	5	0.2%	183	6.0	2.0±0.9
Total	2,678	86.6	413	13.4%	3,091	100	3.0±1.7

SD, standard deviation.

**Table 3:** Determining factors affecting the likelihood of culling within 400 days postpartum using Cox’s proportional hazard model

Variable	The likelihood of culling within 400 days postpartum		

	Hazard ratio1)	95% CI	p-value
Main culling reasons			
Voluntary causes (n = 573)2)	Reference3)		
Infertility (n = 860)	0.28	0.247–0.315	<0.0001
Mastitis (n = 635)	0.85	0.750–0.956	0.0073
Peripartum disorders (n = 435)4)	2.25	1.969–2.574	<0.0001
Other health problems (n = 287)5)	0.86	0.736–0.992	0.0392
Parity			
1 (n = 547)	Reference		
2 (n = 658)	1.13	0.994–1.279	0.0629
3 (n = 594)	1.16	1.018–1.319	0.0253
4 (n = 454)	1.36	1.187–1.560	<0.0001
5 (n = 264)	1.70	1.451–1.995	<0.0001
≥6 (n = 273)	1.73	1.473–2.039	<0.0001
Calving season			
Spring (n = 579)	Reference		
Summer (n = 769)	1.12	0.994–1.254	0.0629
Autumn (n = 687)	0.97	0.858–1.090	0.5816
Winter (n = 755)	1.02	0.910–1.149	0.7105

1) A hazard ratio (HR) of 1 indicates no difference in risk in between groups; an HR greater than 1 indicates an increased risk, and an HR less than 1 indicates a decreased risk.

2) Voluntary causes indicate high parity, low milk yield, bad temperament, high somatic cells, udder problems, and overstock.

3) Reference denotes the baseline category within each variable against which other categories are compared: main culling reasons = voluntary causes, parity = 1, and calving season = spring.

4) Peripartum disorders indicate dystocia, retained placenta, uterine rupture, uterine or vaginal prolapse, septicemic metritis, uterine adhesion, cervicitis, pyometra, ketosis, milk fever, abomasal displacement, and clinical endometritis.

5) Other health problems were indicate, fracture, peritonitis, sinusitis, traumatic reticuloperitonitis, pneumonia, cardiovascular diseases, heat stress, shock, and tumors.

CI, confidence interval.

**Table 4:** Determining factors affecting culling risk by classifying the postpartum period using logistic regression in the GLIMMIX procedure

Variable	Culling risk over time after calving1)								

	Period 1			Period 2			Period 3		
		
	Odds ratio2)	95% CI	p-value	Odds ratio	95% CI	p-value	Odds ratio	95% CI	p-value
BCS at dry-off			<0.0001			0.961			0.491
≤3 (n = 1,676)	0.96	0.691–1.336		-	-		-	-	
3.25 (n = 1,765)	1.08	0.803–1.438		-	-		-	-	
3.5 (n = 5,211)	Reference			-	-		-	-	
≥3.75 (n = 1,198)	1.83	1.403–2.398		-	-		-	-	
Change in BCS3)			0.019			0.012			0.424
No change (n = 2,715)	Reference			Reference			-	-	
Increase (n = 871)	0.65	0.431–0.980		0.98	0.736–1.298		-	-	
Decrease (n = 6,264)	1.17	0.929–1.464		1.29	1.080–1.547		-	-	
Peripartum disorders4)			<0.0001			0.006			<0.0001
No (n = 7,558)	Reference			Reference			Reference		
Yes (n = 2,292)	1.65	1.358–1.994		1.26	1.069–1.473		1.36	1.180–1.566	
Parity			<0.0001			<0.0001			<0.0001
1 (n = 3,537)	Reference			Reference			Reference		
2 (n = 2,595)	0.93	0.654–1.308		2.08	1.623–2.661		1.63	1.328–1.998	
3 (n = 1,768)	1.94	1.408–2.676		2.57	1.993–3.311		1.91	1.543–2.359	
4 (n = 1,023)	2.97	2.125–4.142		3.66	2.794–4.804		2.06	1.619–2.619	
5 (n = 532)	4.28	2.963–6.189		4.25	3.100–5.830		2.25	1.674–3.016	
≥6 (n = 395)	5.69	3.898–8.313		5.86	4.225–8.119		2.87	2.091–3.925	
Calving season			0.244			0.463			0.086

1) Culling risk within 60 days (Period 1), between 61 and 210 days (Period 2), and beyond 210 days (Period 3) after calving.

2) An odds ratio (OR) of 1 indicates no difference in risk in between groups; an OR greater than 1 indicates increased risk, and an OR less than 1 indicates decreased risk.

3) BCS change between dry-off and calving.

4) Peripartum disorders were dystocia, retained placenta, uterine rupture, uterine or vaginal prolapse, septicemic metritis, uterine adhesion, cervicitis, pyometra, ketosis, milk fever, abomasal displacement, and clinical endometritis.

CI, confidence interval; BCS, body condition score.

## Data Availability

Upon reasonable request, the datasets of this study can be available from the corresponding author.
